# Large diameter hemicraniectomy does not improve long-term outcome in malignant infarction

**DOI:** 10.1007/s00415-023-11766-3

**Published:** 2023-05-10

**Authors:** Dominik Lehrieder, Hans-Peter Müller, Jan Kassubek, Nils Hecht, Götz Thomalla, Dominik Michalski, Thomas Gattringer, Katja E. Wartenberg, Jörg Schultze-Amberger, Hagen Huttner, Joji B. Kuramatsu, Silke Wunderlich, Hans-Herbert Steiner, Karin Weissenborn, Suzette Heck, Albrecht Günther, Hauke Schneider, Sven Poli, Christian Dohmen, Johannes Woitzik, Eric Jüttler, Hermann Neugebauer, on behalf of the DESTINY-R (DEcompressive Surgery for the Treatment of malignant INfarction of the middle cerebral arterY - Registry) Study Group and IGNITE (Initiative of German NeuroIntensive Trial Engagement) Study Group

**Affiliations:** 1 Department of Neurology, University Hospital Würzburg, Josef-Schneider-Straße 11, 97080 Würzburg, Germany; 2 Department of Neurology, University Hospital Ulm, Ulm, Germany; 3 Department of Neurosurgery and Center for Stroke Research Berlin, Charité – Universitätsmedizin Berlin, Corporate member of Freie Universität Berlin, Humboldt-Universität Zu Berlin, and Berlin Institute of Health, Berlin, Germany; 4 Department of Neurology, University Medical Center Hamburg-Eppendorf, Hamburg, Germany; 5 Department of Neurology, University Hospital Leipzig, Leipzig, Germany; 6 Department of Neurology, Medical University of Graz, Graz, Austria; 7 Department of Neurology, University of Halle-Wittenberg, Halle/Saale, Germany; 8 Department of Neurology, Klinikum Ernst Von Bergmann Potsdam, Potsdam, Germany; 9 Department of Neurology, University Hospital Giessen, Giessen, Germany; 10 Department of Neurology, University Hospital Erlangen, Erlangen, Germany; 11 Department of Neurology, School of Medicine, Klinikum Rechts der Isar, Technical University of Munich, Munich, Germany; 12 Department of Neurosurgery, Paracelsus Medical University, Nuernberg, Germany; 13 Department of Neurology, Hannover Medical School, Hannover, Germany; 14 Department of Neurology, University of Munich, Ludwig Maximilian University, Munich, Germany; 15 Department of Neurology, University Hospital Jena, Jena, Germany; 16 Department of Neurology, University Hospital Dresden, Dresden, Germany; 17 Department of Neurology, University Hospital Augsburg, Augsburg, Germany; 18 Department of Neurology and Stroke, Eberhard-Karls University Tuebingen, Tuebingen, Germany; 19 Hertie Institute for Clinical Brain Research, Eberhard-Karls University, Tübingen, Germany; 20 Department of Neurology, University Hospital Cologne, Cologne, Germany; 21 Department for Neurology and Neurological Intensive Care, LVR Clinic Bonn, Bonn, Germany; 22 Department of Neurosurgery, University Hospital Oldenburg, Oldenburg, Germany; 23 Department of Neurology, Ostalb-Klinikum Aalen, Aalen, Germany

**Keywords:** Middle cerebral artery infarction, Hemicraniectomy, Functional outcome, Size of hemicraniectomy, Malignant stroke

## Abstract

**Introduction:**

In malignant cerebral infarction decompressive hemicraniectomy has demonstrated beneficial effects, but the optimum size of hemicraniectomy is still a matter of debate. Some surgeons prefer a large-sized hemicraniectomy with a diameter of more than 14 cm (HC > 14). We investigated whether this approach is associated with reduced mortality and an improved long-term functional outcome compared to a standard hemicraniectomy with a diameter of less than 14 cm (HC ≤ 14).

**Methods:**

Patients from the DESTINY (DEcompressive Surgery for the Treatment of malignant INfarction of the middle cerebral arterY) registry who received hemicraniectomy were dichotomized according to the hemicraniectomy diameter (HC ≤ 14 cm vs. HC > 14 cm). The primary outcome was modified Rankin scale (mRS) score ≤ 4 after 12 months. Secondary outcomes were in-hospital mortality, mRS ≤ 3 and mortality after 12 months, and the rate of hemicraniectomy-related complications. The diameter of the hemicraniectomy was examined as an independent predictor of functional outcome in multivariable analyses.

**Results:**

Among 130 patients (32.3% female, mean (SD) age 55 (11) years), the mean hemicraniectomy diameter was 13.6 cm. 42 patients (32.3%) had HC > 14. There were no significant differences in the primary outcome and mortality by size of hemicraniectomy. Rate of complications did not differ (HC ≤ 14 27.6% vs. HC > 14 36.6%, p = 0.302). Age and infarct volume but not hemicraniectomy diameter were associated with outcome in multivariable analyses.

**Conclusion:**

In this post-hoc analysis, large hemicraniectomy was not associated with an improved outcome or lower mortality in unselected patients with malignant middle cerebral artery infarction. Randomized trials should further examine whether individual patients could benefit from a large-sized hemicraniectomy.

**Clinical trial registration information:**

German Clinical Trials Register (URL: https://www.drks.de; Unique Identifier: DRKS00000624).

**Supplementary Information:**

The online version contains supplementary material available at 10.1007/s00415-023-11766-3.

## Introduction

In malignant cerebral infarction space-occupying brain edema is associated with a mortality of up to 80% despite intensive care treatment [4]. Early hemicraniectomy within 48 h of symptom onset has demonstrated improved functional outcome and to reduced mortality in randomized controlled trials (RCTs) [18, 22]. Despite the beneficial effects of this intervention, about 20% of patients still die from herniation in the acute phase after hemicraniectomy, which theoretically might be avoided by a larger hemicraniectomy resulting in additional relief for the swollen brain tissue [8, 22].

The volume gained by hemicraniectomy directly correlates with the diameter of the bone flap. Current recommendations are based on in vitro models which calculated a minimum diameter of 12 cm to create an additional volume of at least 80 ml [28]. While hemicraniectomies with a diameter of less than 12 cm may bear the risk of transcalvarial herniation and hemicraniectomy-associated hemorrhages due to shear injury at the bone edges, larger bone flaps of more than 14 cm in diameter may be associated with more surgical complications such as bleedings, damage to bridging veins or sinus, postsurgical hydrocephalus, and the sinking skin flap syndrome [10, 26]. Several retrospective studies reported controversial data about the association of larger bone flaps with improved functional outcome or mortality, but were all limited by retrospective single-center designs and small sample sizes [1, 6, 11, 14, 20]. Nevertheless, current national and international guidelines for management of large hemispheric infarction recommend a standard diameter of at least 12 cm, and suggest a larger diameters of 14–16 cm with a moderate quality of evidence [21, 24]. Thus, the optimal diameter of hemicraniectomy remains to be defined. The present study used prospectively collected data from a multicenter registry to evaluate long-term functional outcome and mortality as well as safety of an enlarged hemicraniectomy with a diameter of more than 14 cm (HC > 14) compared to a diameter of less than 14 cm (HC ≤ 14).

## Methods

Between January 2010 and July 2016 patients with large ischemic infarction were prospectively included into the DEcompressive Surgery for the Treatment of malignant INfarction of the middle cerebral arterY—Registry (DESTINY-R) study in 30 neurological and neurosurgical departments in Germany and Austria.

A detailed description of the study design has been published previously [15]. The trial was registered in the German Registry for Clinical Studies (DRKS00000624). The ethics committee of the Charité – Universitätsmedizin Berlin, Campus Benjamin Franklin (EA4/108/09) and the local ethics committees of all participating centers approved this registry. Written informed consent was obtained from all patients or legal representatives.

For this analysis patients who fulfilled the following inclusion criteria were selected from the complete study cohort: (1) ischemic infarction of at least 50% of the middle cerebral artery territory confirmed by computed tomography (CT) or magnetic resonance imaging (MRI) with corresponding clinical signs of a severe hemispheric syndrome, with or without additional infarction of the ipsilateral anterior or posterior cerebral artery territory, (2) hemicraniectomy performed according to the decision of the treating physician independent of study participation, (3) available postoperative imaging data measuring hemicraniectomy diameter, and (4) completed follow-up assessment of functional outcome conducted after 12 months. Patients with concomitant acute contralateral and/or infratentorial infarction or additional acute traumatic brain injury were excluded. The timing of hemicraniectomy and choice of the surgical technique (including the diameter of the bone flap, type of duraplasty) were left to the discretion of the treating neurosurgeon with a reference to the current guidelines [21].

After study inclusion, sociodemographic factors, clinical parameters (stroke severity on admission, level of consciousness on admission and before neurosurgery), pre-morbid functional status, vascular risk factors as well as surgical and medical treatment data were documented in a case report form [3]. Additionally, relevant parameters of hemicraniectomy as well as complications of neurosurgery and during intensive care treatment were documented. Follow-up assessment was performed via a structured telephone interview 12 months after stroke onset.

### Clinical outcome analysis

The primary outcome was defined as the functional status according to the modified Rankin Scale score (mRS) after 12 months. Clinical outcome was dichotomized as mRS 0–4 versus 5 and 6 according to the definition of the pooled analysis of the three European hemicraniectomy RCTs [22]. Secondary outcome measures were (1) in-hospital mortality, (2) mortality within 12 months after stroke onset, (3) complications regarding intensive care treatment or neurosurgery and (4) functional outcome dichotomized as mRS 0–3 versus 4–6 [13, 16]. In addition, a subgroup analysis was performed in younger patients ≤ 60 years.

### Neuroimaging analysis

Neuroradiological parameters were processed centrally and blinded to functional outcome on the basis of CT or MRI scans on admission and after neurosurgery using *TIFT* (Tension Imaging and Fiber Tracking) software [12, 17, 19]. After imputation of imaging data all scans were transformed into a three-dimensional grid with a voxel size of 0.5 mm by nearest-neighbour-transformation and the brain was aligned along a horizontal line between the anterior and posterior commissure. For the volumetric measurement of infarct volume at inclusion semi-automatically three-dimensional delineation of hypodense tissue on CT and hyperintensity on diffusion-weighted MRI was used to identify lesion-related voxels. Subsequently, opening and closing procedures were performed to delineate a homogeneous infarct region and to exclude false positive voxels. In order to ensure the comparability of the calculated volumes, infarct volume was standardized for total intracranial volume [27]. In contrast, hemicraniectomy diameter was not standardized, because the neurosurgeons used absolute values as a guide during the operation.

**Fig. 1:**
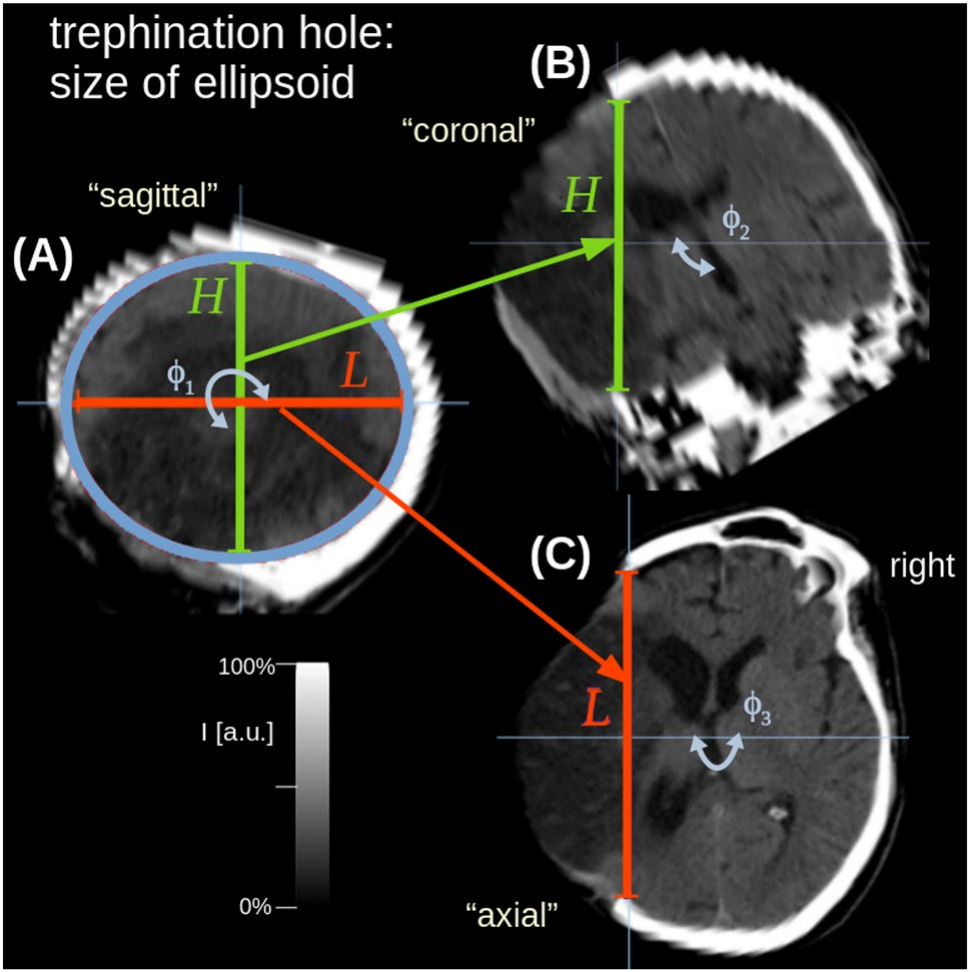
Non-contrast computed tomography CT scan in representative slices after alignment (rotation angles ϕ_1_, ϕ_2_, ϕ_3_) of the trephination hole: **A** “sagittal” slice aligned with the trephination hole as an ellipse for calculation of the surface area (light blue); **B** “coronal” slice with the height of the minor axis (H, green); **C** “axial” slice with the major axis defining the “diameter” of the hemicraniectomy (L, red)

To simplify the measurement of the hemicraniectomy diameter on postoperative imaging data, the bone flap was considered as an ellipse oriented in sagittal axis (see Fig. 1): First, the image showing the largest diameter of the bone flap in the axial plane was selected and aligned along the sagittal axis (larger axis = major). Second, the same procedure was performed in the coronal plane to define the smaller axis (minor). Subsequently, the ellipse was rotated in the sagittal plane to obtain the minor and major axes directly from coronal and axial coordinates (Fig. 1). The diameter of the hemicraniectomy was defined as the length (L) of the ellipse´s major axis, while height (H) was defined as the ellipse´s smaller axis (minor). Surface area (A) of the hemicraniectomy was calculated using the two perpendicular parameters and the following formula: A = L/2 × H/2 × π.

All patients were dichotomized in two groups based on the diameter of the bone flap according to the larger major axis defining diameter ≤ 14 cm as “standard hemicraniectomy” (HC ≤ 14) and > 14 cm as “large hemicraniectomy” (HC > 14) [11, 14, 21]. Additionally, amount of midline shift and involvement of anterior/posterior cerebral artery territory, and uncal herniation at study inclusion were assessed on pre-operative neuroimaging.

### Statistical analysis

Statistics were performed using the SPSS 26 software package (SPSS Inc., Chicago, Illinois, USA). Descriptive analyses were calculated for all variables. Student´s t test, Mann–Whitney U test, or χ^2^ test were used as appropriate to identify differences between the two treatment-groups (HC ≤ 14 vs. HC > 14). To prevent confounding by indication, i.e., performing larger diameter of hemicraniectomy for larger infarctions, linear regression analysis between infarct volume at study inclusion and diameter of hemicraniectomy was performed. Using receiver-operating characteristic (ROC) analysis, threshold levels of hemicraniectomy diameter were calculated to evaluate the cut-off suggested by current guidelines of 14 cm. Diameter of bone flap and parameters of neurosurgery and conservative therapy were entered in a binary logistic regression model to evaluate their impact on functional outcome adjusted for age and infarct volume on admission. Results were expressed as odds ratios with 95%-Confidence Interval (OR, 95%-CI). The level of significance was defined as p < 0.05.

## Results

Among 168 patients with hemicraniectomy, functional outcome data after 12 months and postsurgical imaging were available from 130 patients who were included in our analysis. Of these patients, 42 (32.3%) were female, mean age (SD) was 54.5 (11.0) years, and 94 (72.3%) patients were younger than 60 years at study inclusion. One year after hemicraniectomy 27 (20.8%) patients had a mRS of 0–3 and 79 (60.8%) patients had a mRS of 0–4. 29 (22.3%) patients had died before the 12 months follow-up. Causes of death during hospital stay (N = 15, 11.5%) were transtentorial herniation (N = 9), cardiac arrest (N = 2), pneumonia (N = 1), pulmonary embolism (N = 1), and unknown (N = 2). Causes of death during the follow-up period after discharge were not assessed. Depending on the decision of the operating neurosurgeon 88 (67.7%) patients underwent standard hemicraniectomy (≤ 14 cm) and 42 (32.3%) patients were treated by large hemicraniectomy (> 14 cm). Large hemicraniectomy was performed at 10 out of 30 study centers and 30 out of 42 (72%) cases were performed at 3 study centers. No significant differences in baseline demographic or clinical characteristics were found between the two hemicraniectomy groups (Table 1). Timing of neurosurgery after symptom-onset did not vary between treatment groups [HC ≤ 14: 29 (21–54) hours vs. HC > 14: 31 (21–50) hours, p = 0.799]. Furthermore, there was no difference in the median time interval between imaging and hemicraniectomy (HC ≤ 14: 6 (3–12) hours vs. HC > 14: 4 (2–15) hours, p = 0.372). Volume of infarction before neurosurgery (HC ≤ 14: 244 ± 63 ml vs. HC > 14: 241 ± 52 ml, p = 0.909), left hemispheric infarction (HC ≤ 14: 43.2% vs. HC > 14: 45.0%, p = 0.982) and concomitant involvement of the anterior cerebral artery (HC ≤ 14: 17.2% vs. HC > 14: 14.3%, p = 0.670) or posterior cerebral artery (HC ≤ 14: 4.6% vs. HC > 14: 4.8%, p = 0.967) territory was similar in both groups. Furthermore, there were no differences in the amount of midline shift (HC ≤ 14: 2.9 ± 3.9 mm vs. HC > 14: 2.3 ± 3.1 mm, p = 0.494) or frequency of uncal herniation (HC ≤ 14: 7.1% vs. HC > 14: 10.0%, p = 0.585) before neurosurgery. Medical treatment of elevated intracranial pressure differed regarding osmotic therapy (HC ≤ 14: 44.8% vs. HC > 14: 28.6%, p = 0.077) and muscle relaxation (HC ≤ 14: 26.4% vs. HC > 14: 9.5%, p = 0.027). In the HC > 14 group the diameter of the craniotomy (HC ≤ 14: 12.8 cm vs. HC > 14: 15.8 cm, p < 0.001) and the area of the craniotomy (HC ≤ 14: 94.7 cm^2^ vs. HC > 14: 119.4cm^2^, p < 0.001) were larger than in the HC ≤ 14 group (Table 2).

**Table 1:** Sociodemographic, clinical characteristics and treatment-related parameters in patients treated with standard hemicraniectomy and large hemicraniectomy

	HC ≤ 14 (*N* = 88)	HC > 14 (*N* = 42)	*p* value
Age in years, *mean (SD)*	55.14 (± 9.78)	53.07 (± 13.22)	0.320
Age group, *N* (%)			
≤ 60 years	64 (72.7)	30 (71.4)	0.877
> 60 years	24 (27.3)	12 (28.6%)	
Sex *N* (%)			
Male	58 (66.7)	29 (69.0)	0.787
Female	29 (33.3)	13 (31.0)	
Pre-morbid mRS, *M* (IQR)	0 (0–0)	0 (0–0)	0.679
NIHSS on admission, *M* (IQR)	18 (15–21)	19 (13–22)	0.967
GCS on admission, *M* (IQR)	11 (10–14)	11 (10–14)	0.696
GCS before neurosurgery, *M* (IQR)	6 (3–10)	9 (3–13)	0.103
Atrial fibrillation, *N* (%)	14 (16.1)	8 (19.5)	0.632
Diabetes mellitus, *N* (%)	24 (27.6)	6 (14.6)	0.107
Arterial hypertension, *N* (%)	55 (63.2)	27 (65.9)	0.772
Smoking, N (%)	36 (42.9)	17 (41.5)	0.882
Intravenous thrombolysis, *N* (%)	28 (31.8)	15 (35.7)	0.659
Mechanical recanalization, *N* (%)	18 (20.5)	11 (26.2)	0.463
Diameter of hemicraniectomy in cm, *M* (IQR)	12.8 (12.1–13.3)	15.8 (15.0–16.2)	< 0.001
Surface area of hemicraniectomy in cm^2^, mean (± SD)	94.7 (13.6)	119.4 (13.9)	< 0.001
Osmotic therapy, *N (*%)	39 (44.8)	12 (28.6)	0.077
Analgosedation, *N* (%)	73 (83.9)	36 (85.7)	0.791
Muscle relaxation, *N* (%)	23 (26.4)	4 (9.5)	0.027
Induced hypothermia,* N* (%)	10 (11.5)	3 (7.1)	0.442

*HC > 14* large hemicraniectomy, *GCS* Glasgow Coma Scale, *IQR* Interquartile Range, *M* Median, *mRS* modified Rankin Scale, *N* Number, *NIHSS* National Institutes of Health Stroke Scale, *SD* Standard Deviation, *HC ≤ 14* standard hemicraniectomy

**Table 2:** Primary and secondary outcome measurement in all hemicraniectomy patients and in the subgroup of patients ≤ 60 years

All patients (*N* = 130)	HC ≤ 14 (*N* = 88)	HC > 14 (*N* = 42)	*p* value
mRS ≤ 4 after 12 months, *N* (%)	53 (60.2)	26 (61.9)	0.855
mRS ≤ 3 after 12 months, *N* (%)	22 (25.0)	5 (11.9)	0.085
In-hospital mortality, *N* (%)	10 (11.4)	5 (11.9)	0.928
Mortality before 12 months, *N* (%)	20 (22.7)	9 (21.4)	0.868

Subgroup of patients ≤ 60 years (*N* = 94)	HC ≤ 14 (*N* = 64)	HC > 14 (*N* = 30)	
mRS ≤ 4 after 12 months, *N* (%)	40 (62.5)	19 (63.3)	0.938
mRS ≤ 3 after 12 months, *N* (%)	20 (31.3)	4 (13.3)	0.063
In-hospital mortality, *N* (%)	9 (14.1)	3 (10.0)	0.582
Mortality before 12 months, *N* (%)	17 (26.6)	6 (20.0)	0.490

*HC > 14* large hemicraniectomy, *mRS* modified Rankin Scale, *N* Number, *HC ≤ 14* standard hemicraniectomy

**Fig. 2:**
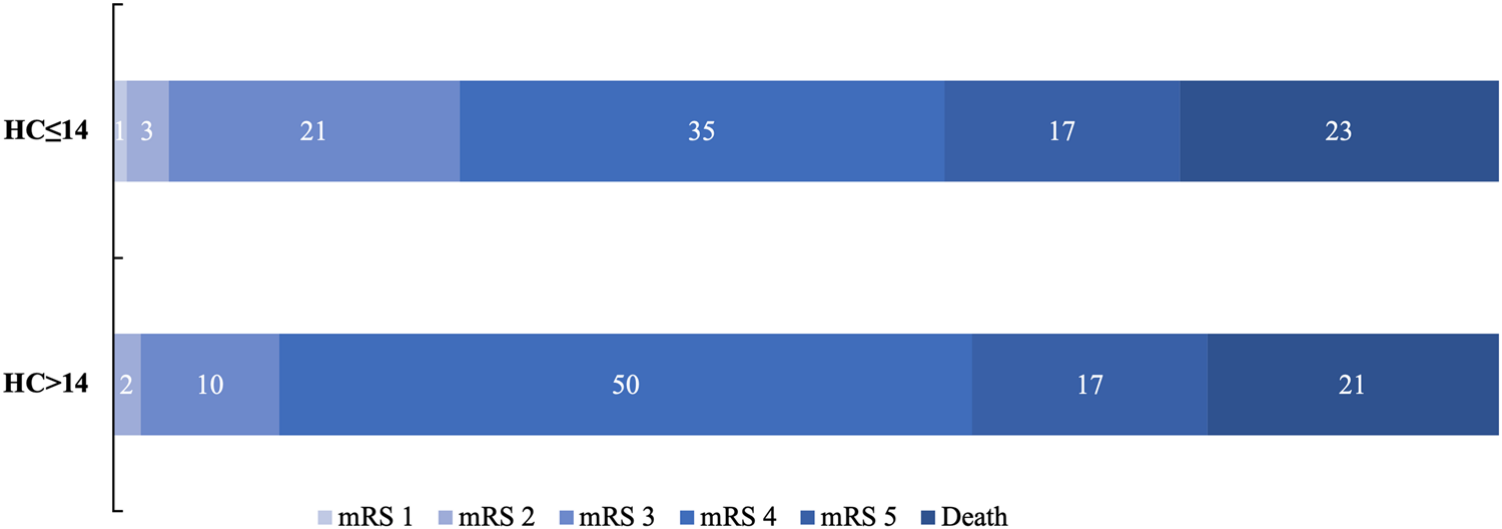
Distribution of the scores on the mRS and deaths (%) after 12 months in patients treated with standard (HC ≤ 14) and large (HC > 14) hemicraniectomy

Table 2 shows the primary and secondary clinical outcome measures. There was no significant difference for the primary endpoint, i.e., mRS 0–4 after one year, between both treatment groups (HC ≤ 14: 60.2% vs. HC > 14: 61.9%, p = 0.855). Furthermore, neither in-hospital mortality nor mortality after 12 months follow-up period differed between both groups. In the HC > 14 group five of 42 patients (11.9%) achieved a mRS 0–3 at follow-up compared to 22 of 88 patients (25.0%) in the HC ≤ 14 group (p = 0.085). Similar results were also found in the subgroup of younger patients (≤ 60 years) and after exclusion of patients with hemicraniectomy after more than 48 h, respectively. Figure 2 illustrates the distribution of mRS scores after 12 months.

In linear regression analysis no association of infarct volume and diameter of bone flap was observed (F(1,118) = 0.216, p = 0.643), suggesting an absence of confounding by indication and the unselected use of large over standard hemicraniectomy. In addition, ROC analyses did not determine a certain hemicraniectomy diameter other than 14 cm that could predict outcome (mRS 0–4: AUC 0.53, 0.42–0.6, p = 0.612; mRS 0–3: AUC 0.46, 0.34–0.57, p = 0.482), in-hospital mortality (AUC 0.50, 0.35–0.65, p = 0.965), or mortality after 12 months (AUC 0.50, 0.38–0.61, p = 0.946) with a sensitivity or specificity higher than chance.

Neurosurgery-related parameters and complications regarding hemicraniectomy or intensive care are shown in Table 3. Type of duraplasty differed between treatment groups: if an enlarged hemicraniectomy was performed, the dura was more often rapidly closed and less often sewed. A similar number of patients experienced at least one complication related to neurosurgery (HC ≤ 14: 27.6% vs. HC > 14: 36.6%, p = 0.302), while the rate of patients with multiple complications was significantly higher after large hemicraniectomy (22.0% vs. 3.4%, p = 0.003). Although wound healing disorders [6 (14.6%) vs. 2(2.3%), p = 0.007] and subdural hematomas [5 (12.2%) vs. 2 (2.3%), p = 0.034] were more frequently observed after a large hemicraniectomy, these differences were small in terms of absolute numbers and observed in only few of the study centers. Furthermore, complications regarding hemicraniectomy equally often required another surgical intervention in both groups (HC ≤ 14: 5.7% vs. HC > 14: 9.8%, p = 0.408). No differences were found between treatment groups concerning duration of mechanical ventilation or frequency of intensive care-associated complications, e.g. pneumonia or deep vein thrombosis. Complications regarding cranioplasty, which was performed after a median of 116 (IQR 90–155) days, were more frequent in the HC > 14 group (70.6% vs. 44.1%, p = 0.012) and required more often another surgical intervention after HC > 14 (32.4% vs. 14.9%, p = 0.041).

**Table 3:** Surgical characteristics, frequency of complications regarding hemicraniectomy, cranioplasty and intensive care

	HC ≤ 14 (*N* = 88)	HC > 14 (*N* = 42)	*p* value
At least one complication due to neurosurgery, *N* (%)	24 (27.6)	15 (36.6)	0.302
Number of complications, *N* (%)			
1 complication	21 (24.1)	6 (14.6)	0.003
2–4 complications	3 (3.4)	9 (22.0)	
Further neurosurgery required, *N* (%)	5 (5.7)	4 (9.8)	0.408
Technique of duraplasty			
Point fixed duraplasty,* N* (%)	9 (10.6)	3 (8.1)	0.672
Sewed duraplasty,* N* (%)	22 (25.9)	4 (10.8)	0.062
Rapid closure duraplasty,* N* (%)	38 (44.7)	26 (70.3)	0.009
Unknown, *N* (%)	16 (18.8)	4 (10.8)	0.272
At least one complication regarding intensive care, *N* (%)	61 (70.1)	31 (75.6)	0.519
Pneumonia, *N* (%)	48 (55.2)	19 (46.3)	0.351
Duration on ICU, *M* (IQR) in days	15 (8–25)	12 (7–18)	0.081
Duration of mechanical ventilation, *M* (IQR) in days	9 (5–16)	11 (3–16)	0.842
Discharged with mechanical ventilation, *N* (%)	30 (34.5)	19 (45.2)	0.238

	HC ≤ 14 (N = 69)	HC > 14 (N = 34)	
Time from craniectomy to cranioplasty in days, *M (*IQR)	113 (90–150)	126 (94–172)	0.388
At least one complication regarding cranioplasty, *N* (%)	30 (43.5)	24 (70.6)	0.012

*HC > 14* large hemicraniectomy, *ICU* Intensive care unit, *IQR* Interquartile range, *M* Median, *N* Number, *HC ≤ 14* standard hemicraniectomy

In multivariable binary regression analyses with outcome as dependent variable, adjusted for age, volume of infarction, number of complications, frequency of wound healing disorders, frequency of subdural hematomas, differences in conservative therapy, and type of duraplasty performed, the diameter of hemicraniectomy was no significant predictor of outcome (Table 4). The additional inclusion of frequency and number of complications regarding cranioplasty had no impact on the results.

**Table 4:** Prediction of functional outcome in multivariable binary regression analysis

	Crude OR (95% CI)	*p* value	Adjusted OR^a^ (95% CI)	*p* value
Acceptable outcome (mRS 0–4)				
Infarct volume, per 10 ml increase	0.886 (0.825–0.961)	0.002	0.886 (0.825–0.961)	0.002
Diameter of hemicraniectomy, per 1 cm increase	0.985 (0.805–1.206)	0.886	–	–
Favorable outcome (mRS 0–3)				
Infarct volume, per 10 ml increase	0.784 (0.700–0.886)	< 0.001	0.761 (0.651–0.877)	< 0.001
Age, per 1 year increase	0.934 (0.895–0.975)	0.002	0.947 (0.900–0.996)	0.035
Diameter of hemicraniectomy, per 1 cm increase	0.853 (0.656–1.110)	0.236	–	–

*CI* Confidence interval, *mRS* modified Rankin Scale, *OR* Odds ratio

^a^Presenting the results of logistic regression using backward selection models, testing the following variables: age, volume of infarction, diameter of hemicraniectomy, number of complications regarding hemicraniectomy, frequency of wound healing disorders and subdural hematomas, conservative therapy (osmotherapy, muscle relaxation) and type of duraplasty (sewed or rapid closure)

## Discussion

Despite early prophylactic hemicraniectomy almost 20% of patients who suffer a malignant middle cerebral artery infarction still die. Most deaths occur early and are due to herniation which raises the question if some patients might benefit from a larger hemicraniectomy or if a larger hemicraniectomy in general could be beneficial [2, 4]. None of the hemicraniectomy RCTs provided data on the size of craniectomy or subgroup analyses concerning this issue. Only few data from observational studies are available comparing different sizes of craniectomy. Current guidelines recommend a diameter of the bone flap of at least 12 cm [24]. The results of the current post-hoc analysis from a prospective, multicenter registry could not demonstrate a benefit from a large hemicraniectomy regarding mortality or long-term functional outcome compared to a standard hemicraniectomy in unselected patients with malignant infarction. Furthermore, the data analyses could not determine a certain hemicraniectomy diameter that was meaningfully associated with any of the prespecified outcomes. However, these observations need to be interpreted with caution due to the low percentage of patients treated with large hemicraniectomy at only a few of the participating centers, and due to the post-hoc approach of this analysis.

Mortality in this study population was similar compared to patients treated with hemicraniectomy in the pooled analysis of RCTs in younger patients with malignant infarction [22]. Functional outcome on the other hand was worse in the present study. This finding is most probably due to the broader inclusion criteria of this multicenter registry, which compared to the RCTs better reflects the real-world demographics by including patients with higher age. This is further supported by findings of a recent patient-level meta-analysis [18].

Since infarct volume can predict outcome after decompressive hemicraniectomy [5], the association of a larger hemicraniectomy diameter and outcome in malignant infarction seems intuitive but so far has previously only been evaluated in six retrospective single center studies, which showed heterogenous results. In accordance with our results, three of these studies did not find an association of the size of craniectomy diameter with functional outcome, physical disability, or quality of life [2, 20, 25]. However, the number of patients included in our study was even larger. Two other studies reported potential benefits of larger hemicraniectomies. One study from 2011 found a significantly higher rate of patients with a mRS ≤ 3 in patients with a larger hemicraniectomy diameter. Here, a large landmark-based craniectomy with a mean diameter of 14.9 (± 0.9) cm was performed in 11 patients compared to a historical control of 13 patients following hemicraniectomy with a bone flap of a mean diameter of 12.6 (± 0.9) cm [1]. Another study from 2019 suggested a benefit in functional outcome (mRS ≤ 3) following a so called maximum hemicraniectomy with a diameter of more than 14 cm including resection of the temporalis muscle with its fascia and an expansive duraplasty in 14 younger patients with malignant infarction compared to outcome data from the literature [11]. However, considering not only the younger patients but the whole study cohort including 11 other elderly patients, only 36% of patients had a mRS ≤ 3 which is both comparable to the results of the latest meta-analysis of individual patient data (37%)[18] as well as to the results in the hemicraniectomy group of the current study (31%). In line with another study from 2016 with 97 patients following hemicraniectomy for malignant infarction, we did not observe a reduction in in-hospital mortality [14].

In RCTs, the rates of hemicraniectomy-related complications were comparatively lower (9% and 7%, respectively) [7, 23], than in observational studies, which show similar rates of complications as our study [9]. Although the complication rate was higher in patients treated with larger hemicraniectomy, we could not confirm an association of hemicraniectomy-associated complications and worse functional outcome with the larger diameter. Furthermore, studies reporting low complication rates and improved outcome following enlarged hemicraniectomy were conducted by experienced neurosurgeons in highly specialized centers and retrospectively evaluated [1, 11, 14].

In our cohort osmotherapy and muscle relaxation were applied more frequently in the standard hemicraniectomy group. Although both treatments had no impact on functional outcome in multivariable analyses, the increased use of osmotherapy and muscle relaxation in patients with smaller hemicraniectomies may be indicative of a poorer control of intracranial pressure compared to patients with larger bone flaps.

Despite the strengths of a prospective multicenter registry, our study is not without limitations: it is a non-randomized study and the decision of timing, diameter, and technique of the hemicraniectomy was left at the discretion of the treating neurosurgeon. Hemicraniectomy with duraplasty as a procedure was not standardized [10], neither were pre- and postoperative treatment algorithms, intensive care monitoring, extubation strategies, or the additional use of intracranial pressure lowering treatments including therapeutic hypothermia. Although this approach bears the risk of confounding, we did not observe differences in any of the clinical or radiological parameters used to determine the indication for hemicraniectomy such as midline shift, time since symptom onset, loss of consciousness, or stroke severity. Furthermore, because this is assumed to be standard neurosurgical practice, we did not evaluate an appropriate decompression of the temporal bone in the registry. However, uncal herniation after surgery was less frequent in both treatment groups, suggesting a comparable temporal bone decompression. Most importantly, hemicraniectomy diameter was also not associated with infarct volume which refutes the assumption that larger infarcts were treated by larger hemicraniectomies. In addition, through the inclusion of a wide range of university and community hospitals, treatment of malignant infarction was carried out by physicians with different clinical and surgical experience. Alternatively, the surface area could be calculated by automated segmentation tools; however, we decided to calculate the bone flap by manual approximation as an ellipse by manual approximation in order to obtain a visual control of the results. The surface area could then be calculated from the semi-major axis. The differences result from an easily determined parameter (diameter) that the treating neurosurgeon has at hand intraoperatively, whereas the determination of the surface area is a parameter which is difficult to reproduce intraoperatively. Finally, we did not assess the rate of application of advanced directives resulting in care limitations despite hemicraniectomy which may impact outcome [29].

In conclusion, our results do not provide evidence that large hemicraniectomy is beneficial regarding functional outcome or mortality as compared to standard hemicraniectomy in unselected patients with malignant middle cerebral artery infarction treated by hemicraniectomy. Although our results indicate that standard hemicraniectomy may be associated with less complications and is sufficient in most cases, selected patients may still benefit from a larger diameter hemicraniectomy to achieve the best possible outcome. This relevant question should be addressed in a randomized controlled trial.

## Supplementary Information

Below is the link to the electronic supplementary material.

Supplementary file1 (DOCX 32 KB)

## Data Availability

Original data are available and will be shared by request from a qualified investigator.
